# After-Care Association for the Insane

**Published:** 1902-10-18

**Authors:** 


### AFTER-CARE ASSOCIATION FOR THE INSANE.

The usefulness of this Association is beyond question. It has a great deal to do and it does it well, but if it is to extend its *clientèle* a large addition to its income is essential. Last year it received in subscriptions and donations the sum of £569, being about £30 more than the previous year. The council had to consider applications made to it by 214 discharged patients, which was 19 in excess of the numbers for the year before.

There are a great many patients discharged as recovered from our county asylums every year, and it often happens that these poor people are quite friendless. There is no one to lend a helping hand. In other cases the friends are willing but unable. The result is that some of these patients who might have remained well for years, or for life, relapse and have to be again certified insane for return to the asylum. It is these cases which the Association helps; and its duties are of a very complex nature. It is not the mere voting of funds with which it concerns itself. It certainly has to find the money, but it has also to investigate the conditions of the patients; it has to find suitable cottages in which to board the cases, and above all it has to be assured that the cottagers are suitable for the care of those entrusted to them. For other cases suitable employment has to be secured, for it is not an idle life that will prevent relapse, but the union of constant occupation with cheerful surroundings and unselfish care. The Council does not shirk its work, and we hear that, wherever possible, those who are willing to employ discharged patients are previously interviewed, and that as many as twenty visits have been made in connection with a single case.

Attention is drawn to the fact that it has been found necessary to increase the payments made to cottagers for maintenance, and hence that unless increased subscriptions or donations are forthcoming, the usefulness of the Association will be crippled.

Annual subscriptions of 5s. constitute ordinary membership, and a single donation of 50s. constitutes life membership. It is a pity that more people would not follow the example set by Mrs. Kendzior, of Purley. This lady has for five consecutive years given an entertainment on behalf of the Association, and on each occasion a considerable sum has been handed to the Council.

Gifts of clothing would be acceptable. The hon. treasurer is Dr. Savage, of 3 Henrietta Street, and the secretary is Mr. H. T. Roxby, of Church House, Dean's Yard, Westminster.

